# To advance sleep science, let’s study change

**DOI:** 10.1093/sleep/zsaf155

**Published:** 2025-06-13

**Authors:** Katharine C Simon, Katherine A Duggan

**Affiliations:** Department of Pediatrics, School of Medicine, University of California Irvine, Irvine, CA, United States; Pulmonology Department, Children’s Hospital of Orange County (CHOC), Orange, CA, United States; Department of Psychology, North Dakota State University, Fargo, ND, United States

**Keywords:** behavioral sleep medicine, public health, aging, research design, statistics, longitudinal studies, data analysis, multivariate analysis, causality

## Abstract

Sleep is critical for physical, cognitive, and mental health, but how sleep supports these domains likely fluctuates across the lifespan. While traditional observational and experimental study designs—often cross-sectional or limited (two-wave) longitudinal designs—have provided valuable insights, they fall short of capturing the dynamic nature of sleep and its effects over time. To fully understand these complex and evolving relationships, multimethod, multi-time point longitudinal designs are required. These approaches can illuminate the temporal dynamics of sleep and its outcomes, offering stronger and potentially causal conclusions. In this article, we aim to empower sleep scientists, clinicians, and trainees with research methods focused on studying change—methods that can be applied across both observational and experimental designs. To truly advance the field, it is critical to examine sleep throughout the lifespan, from infancy through older adulthood, with repeated and nuanced assessments of sleep and its related outcomes. We outline a variety of statistical analysis approaches and corresponding design considerations that support the rigorous study of change in sleep. Finally, we offer forward-looking recommendations for scientific training, research program evaluation and funding, and the development of research infrastructure and collaborations. Together, these strategies have the potential to propel the field of sleep research forward, generating richer insights and change-based conclusions.

**Statement of Significance**

As a field, we are driven by a fundamental question: does sleep temporally precede and cause changes to our physical, mental, and cognitive health? While many existing studies use cross-sectional or limited (two-wave) longitudinal designs, these approaches often fall short of capturing the full picture needed to understand the timing and impact of sleep. Encouragingly, we have the tools and methods needed to pursue this important work. In this article, we highlight statistical approaches and research designs that can help move the field forward. With thoughtful application of these methods, we can strengthen our conclusions, generate more impactful findings, and bring us closer to understanding the role of sleep across the lifespan.

## To Advance Sleep Science, Let’s Study Change

### Sleep matters

Sleep scientists and allies have excelled at demonstrating the need for healthy, restorative sleep across the lifespan [1–4]. Decades of research—summarized in consensus statements, systematic reviews, and meta-analyses—have shown that sleep is related to an array of consequential outcomes, including but not limited to academic achievement [5], cognitive function [6], safe driving [7], pain [8], psychiatric disorders [9], systemic inflammation [10], obesity [11], diabetes [12], cardiometabolic health [13], dementia [14], and all-cause mortality risk [14]. Sleep is also related to social determinants of health [15, 16], including socioeconomic status [17], race/ethnicity [18], and discrimination [19]. Finally, inadequate sleep is costly; insufficient sleep costs the United States over 1 million working days annually [20], and total financial costs are more than 1.5 per cent of the Australian gross domestic product [21].

This rich and expansive body of literature consistently highlights the importance of sleep. However, to truly advance our understanding, we must move beyond broad statements such as “sleep matters” to uncovering *when*, *how*, and *why* sleep matters—something that current study designs often struggle to address. Most sleep research relies on observational, cross-sectional, limited (two-wave) longitudinal cohort designs, as well as single-night experimental studies. While these approaches provide valuable insights, they do not carefully capture the dynamic, fluctuating temporal nature of sleep’s relationship with health and cognition. Without the index of time, when associations are assessed at a single time point—such as in the case of cross-sectional designs—there is no way to determine directionality or whether associations are potentially causal or due to a third, possibly spurious variable [22, 23]. Similarly, in simple two-time point longitudinal studies, change is often assumed to be linear and static (i.e. the same for all people across all ages and contexts), discounting the possibility of different rates of change over time. In these cases, what appears to be change might instead reflect measurement error or some other erroneous conclusion [24]. In experimental designs, within-subject studies can probe specific mechanisms—such as the role of spindles [25]—but whether these mechanisms vary across critical life periods is unknown. While many studies adjust for a range of demographic, psychosocial, behavioral, physiological, and cognitive factors, these research designs do not fully capture the temporal progression of these factors or how their change over time is related to changes in sleep over time.

To capture change, multiple time points of the predictor and outcome must be measured. Even more importantly, to draw causal conclusions, change in the predictor must precede change in the outcome. Most two-wave longitudinal designs measure the predictor and the outcome at only one or two time points, making it challenging to establish directionality and therefore causality. Instead, by indexing time across three or more time points, and anchoring those time points to meaningful, hypotheses-driven factors, we can begin to ask richer and more nuanced questions: How does this relationship change over time? Does one factor precede changes in another? What patterns of change emerge over time? In doing so, we can shift towards understanding the structure and meaning of change. Time can be indexed in ways that reflect change in an individual’s status (e.g. their age or pubertal status) or as a function of an intervention (e.g. the number of therapy sessions). From here, we can gain insight into the underlying temporal relationships, getting at how the relationship between sleep and health changes over time, and how one factor may precede or lag behind changes in another. Developing an understanding of these longitudinal dynamics across multiple time scales, from moment-to-moment to year-to-year, is key to identifying life-span mechanisms relevant to sleep.

## Why Study Change?

**Figure 1:**
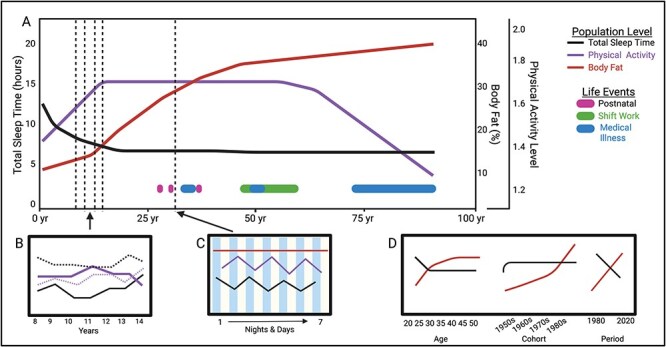
(A) Graphical abstract illustrating longitudinal trajectories of sleep duration, physical activity, and body fat [26]. Within- and between-person trajectories are depicted based on previous publications whenever possible [27–30]. Examples of key life events are shown at the bottom of panel (A), representing potential influences that could be incorporated into longitudinal research designs. (B) The annual relationship between sleep (black) and physical activity (purple). Solid lines represent group-level averages, while dashed lines illustrate an example individual’s data. (C) Example of a 7-day consecutive data collection approach. Light columns represent days and dark columns represent nights. A cross-sectional study using only one or two time points might incorrectly conclude there is no relationship between the three variables. However, with three or more time points, a clearer pattern emerges, revealing day-to-day coupling of sleep and physical activity. (D) Example of evaluating sleep duration and body fat by age (tracking an individual over time), by cohort (comparing generational changes), or by period (evaluating historical influences).

*If we study change, what do we gain*? The answer is: a powerful lens into how sleep’s role evolves across the lifespan. By using robust, truly longitudinal designs, we can begin to unravel challenging and complex public health, cognitive, or aging issues. Studies that include multiple assessments of sleep and outcomes allow us to draw conclusions about the *directionality* of these associations, which is crucial for understanding how sleep influences key outcomes. We highlight several of these considerations in Figure 1 and in the discussion below, using examples from sleep and obesity research, as well as broader methodological decisions.

### The example of sleep and obesity research

The association between short sleep duration and obesity in children and adults is well-documented [31, 32], but to translate our research findings into public health solutions, we need to understand the directionality of this relationship. Guided by insights from statistics, public health, epidemiology, and the social sciences [22, 33–42], several plausible theoretical models emerge. For example, could the association between sleep duration and obesity be spurious, driven by a third variable, such as a gene which predisposes people to short sleep durations and increased health risk [43]? Or might there be reverse causation at play, as people with worse health status in general tend to be overweight and they are more likely to experience worse sleep [44]? Or is the association *truly* causal—where short sleep durations lead to changes in health-relevant mechanisms such as stress, inflammation, or physical activity—which in turn predisposes people to become overweight [45, 46]?

It is equally important to recognize an important corollary to these three models: interventions aimed at reducing the prevalence of short sleep duration will only yield meaningful public health benefits if the association is truly causal. Given the range of possibilities, the key question is: How can we confidently determine directionality? To answer this, our study designs and methods must align with the conclusions we hope to draw, but unfortunately, many current approaches fall short. For example, most observational research on sleep duration and obesity has limited follow-up—with longitudinal studies in children averaging a median follow-up of 4.25 years [32] and those in adults averaging 6.5 years [31]. Moreover, many studies measure sleep and obesity at only one time point each, making it impossible to clearly distinguish whether an observed association that appears prospective and potentially causal might actually be spurious or reverse causal. To establish true causality, studying *change over time* is essential. However, we cannot meaningfully study change without at least three time points. By increasing the number of observations over time, we can capture the evolution of these relationships.

Lastly, observational studies of adults in particular can be limited because many adults already live with chronic illness. Over half of American adults have one diagnosed chronic illness [47], a quarter of American adults have two or more diagnosed conditions [48], and rates of undiagnosed disease are undoubtedly higher. As many sleep and obesity studies focus on midlife adults in their 40s to 60s, it can be especially challenging to untangle these pathways and eliminate the possibility of reverse causation, particularly when chronic illness is already a factor [22, 37]. Aging and chronic illness can manifest through sleep disturbances, highlighting the importance of precise measurement in uncovering the true nature of these relationships. By incorporating reliable and valid repeated measurements of multiple constructs in the nomological network related to sleep and obesity, researchers can gain insight into how these constructs evolve over time. With multiple time points, we can detect meaningful patterns, distinguish stability—and thus, time-based dependencies—in constructs across time. With enough measurements, previously unmeasured systematic error can more easily be identified and ascribed to other factors—including underlying health conditions not readily apparent. By embracing repeated, longitudinal measurements of each construct of interest, as well as the proximal and distal factors in their etiology, we can identify if an unknown underlying health condition is influencing (confounding) the relationships of interest.

Experimental designs, including manipulations of sleep and its mechanisms, are powerful tools for generating causal conclusions [49, 50], and they are widely used in our field. For example, we have developed innovative manipulations of sleep timing, architecture, duration, fragmentation, and physiologic events [25, 51]. Studies that experimentally shorten sleep duration for brief periods of time have revealed changes in leptin and ghrelin, hormones regulating hunger and satiety [52, 53]. These studies provide some evidence for a temporal, *causal* relationship between sleep and obesity, and add mechanistic insight into this literature. However, even these experimental studies have their own limitations and cannot address every challenge described above. Critically, experimental manipulations, timescales, and samples used to generate causal conclusions may lack ecological, real-world validity compared to the types of associations that are likely unfolding at the larger, population level or over longer time periods that span critical age ranges or life transitions.

First, for ethical reasons, experimental studies are often limited to relatively short manipulations of sleep duration, and most participants in these studies are intentionally selected to be healthy and free of chronic or acute health conditions. Second, some manipulations—such as sleep extension for short sleepers—are more feasible and ethical than other manipulations—such as sleep extensions for moderate sleepers or long-term sleep deprivation studies. Third, many of these studies measure surrogate endpoints (e.g. behavioral or physiological mechanisms), rather than measure obesity itself; doing so would be practically impossible as body weight is unlikely to grossly change within a 1-week or less time frame. Because of these constraints, the use of these endpoints can lead to problems of extrapolation and inference, particularly when the mechanism is not well understood and when biomarkers are challenging to reliably and precisely measure. These problems are most likely to occur with only two time points, where regression to the mean can create an illusion of change even when there is no “true” change (i.e. change that is not confounded with measurement error [54, 55]). In extreme cases, this issue can lead to a misleading (opposite) inference, relative to what may occur at the population level [56]. While these experimental studies remain invaluable for advancing mechanistic insight and suggest sleep causes changes in hormone mechanisms in short time frames, they cannot definitively close the case on whether sleep duration causes obesity over longer periods of time at a population level.

Other related fields have made significant strides in addressing similar challenges. For instance, in public health, epidemiologists use methods like matched case–control designs [57] and propensity scores [42] in the design of their studies to aid in estimates of causal effects. However, most studies of sleep are not large-scale epidemiological studies designed to test hypotheses about sleep specifically. Instead, these studies incorporate sleep as a covariate, and thus lack the matching required to generate targeted strong conclusions about sleep.

Although these studies are small snapshots of research on sleep and consequential outcomes, the themes are clear: our current designs limit our ability to confidently establish that sleep temporally precedes and causally influences these outcomes. However, by shifting toward change-based designs, we can significantly enhance our ability to draw directional (and ideally causal) conclusions, thereby advancing our science and its potential to improve public health. One of the most effective ways for our field to generate temporally ordered conclusions consistent with causality is to fully leverage longitudinal designs. *To advance the field, we need to study sleep across the entire lifespan—from infancy to old age—repeatedly assessing sleep and its downstream effects.*

## Starting with the Basics: Guiding Principles in the Study of Change

Understanding the stability and change in sleep and its consequences is key to advancing our field. To draw meaningful conclusions about the consequences of different sleep patterns across the lifespan, longitudinal methods focused on change are required [58, 59]. Excitingly, these methods can be adapted to explore two types of longitudinal change: within-person change (i.e. intra-individual change, examining how each person changes over time) and between-person change (i.e. interindividual change, exploring the factors that predict differences in people and their rates of change [60]). Developmental psychologists, methodologists, and biostatisticians agree that several design considerations must be addressed in research designs that study change [60–63]. Below, we detail how these elements can be effectively incorporated into sleep research. While comprehensive studies of change in sleep would ideally include all essential elements, we acknowledge that practical constraints can present challenges, limiting the feasibility of researchers to fully enact these components. Nevertheless, even small steps towards adopting any of these principles will propel our science forward, enhancing our ability to make precise and impactful claims about the role of sleep in consequential outcomes, including health. Toward the end of this article, we also offer practical and actionable steps to help incorporate longitudinal methods into research designs, empowering researchers to begin making meaningful progress.

### Timing matters: connect your research design to your hypotheses

The temporal dynamics of research designs have critical implications for the conclusions that can be drawn. One essential element is how time is defined. Sleep research is often designed where data collection intervals are spaced apart by weeks, months, or even years. If time in these designs uses measurement wave as the index (i.e. calling data collected at wave 1 “Time 1,” data collected at wave 2 “Time 2,” and so forth), this tells the model that any changes are tied explicitly to the *wave*, rather than some other measure (e.g. participant age, number of therapy sessions, or elapsed time since a particular event). Without any other additional measures of time (e.g. age, or the time lag between assessments), the analysis will also assume measurement waves are spaced equally apart (e.g. the distance between waves 1 and 2 is the same as the distance between waves 2 and 3). In contrast, when data are collected at specific ages, or when time is indexed using age, conclusions can be drawn about the potential underlying developmental processes [64]. Regardless of whether data collection schedules are fixed or flexible, and regardless of whether they are collected across days or years [65, 66], time can be re-conceptualized using chronological age, so long as the temporal data on age are collected in advance. Therefore, we recommend researchers collect a range of time-related metrics a priori, rather than attempting to recreate them post-study. At a minimum, we suggest recording the exact date of each assessment, as well as participants’ birth date (at least in terms of month and year; the exact day of birth can be set to the 15th of the month for all participants, if needed for participant privacy). This will allow for accurate calculation of chronological age at each measurement occasion.

### Timing matters: three or more measurement occasions are required to study change

A second, critical aspect of developmentally informed longitudinal designs involves the total number of measurements. To analyze change in the developmental sense, at least three waves of data are required [60, 67]. This is because developmentalists statistically and methodologically separate several influences on and contexts for individuals’ lives, including age (i.e. maturation due to time, social experience, physiological changes, or a combination thereof), cohort (i.e. differences between groups of people, typically tied to birth year), and period effects (i.e. variations over time that influence all age groups simultaneously, including changes in the environment and historical events/exposures [62, 68–70]). It is not practical or possible to tease apart all three of these influences in a single study. However, the biggest issue for sleep science is the separation of age and cohort, in part because period is often correlated with cohort, and also because research designs often compare groups of people who differ on age.

Typical cross-sectional studies confound age, cohort, and period. In these designs, data are collected at one time point; even if a study includes multiple groups of people that differ in age, the groups will also differ in cohort and period. For example, if a group of emerging adults (ages 18–29) seems different from people in late life (ages ≥ 60) at a single time point, there are a number of potential alternative explanations: any “differences” that seem due to age could also be due to differences in cohort (e.g. generational differences), period (e.g. being alive during and remembering 9/11), or even measurement error (e.g. if the measure has more reliability or validity in one population relative to the other). A natural extension of this model is to follow both groups at least twice each, in a simple two-wave longitudinal study. Here, “change” is indexed using the difference in scores for each group (i.e. T2 − T1). However, this tells us nothing about the shape of the change—whether it is linear, quadratic, or something else. The pattern of change is important because it provides insight into the underlying causal processes and intervention targets. Critically, two-time point studies confound change with measurement error; regression to the mean is common, even in the absence of “true” change [24, 71, 72]. As a result, any observed differences between time points in a two-wave study could reflect measurement error. Furthermore, when only two cohorts are studied, age-related changes might instead be indexing cohort-related differences in measurement error.

Examples of the perils of confusing age and cohort are common in the cognitive developmental literature. One well-known example is the Flynn effect [73]: later (more recent) cohorts score higher on intelligence quotients (IQs) tests, on average, than earlier cohorts, necessitating re-norming of IQ tests across successive cohorts. Another well-known example involves cognitive abilities from the multi-decade Seattle Longitudinal Study: when compared at the same age, later born (younger) cohorts score better and decline less steeply on cognitive tests than earlier born (older) cohorts [74]. However, some of these differences are attributable to selection effects; when models account for terminal decline (i.e. changes in cognition present 3 years prior to death) in both cohorts, some cohort differences become non-significant or even reverse [75]. Some possible study designs that do not confound age and cohort effects include cohort-sequential designs [70, 76]; recruiting additional cohorts in future waves of the study allows for the disentangling of age- and cohort-related differences. When this is not feasible, one can acknowledge that the age- and cohort-related differences are confounded as a limitation in the discussion section.

With at least three measurement occasions for each variable, we can separate true linear change from measurement error. From the perspective of degrees of freedom (*df*s): 2 *df*s are available with three time point data (because *df* = time points − 1). One of these is used to estimate measurement error, and the remaining is used to estimate linear change. Thus, a general rule is to include at least one more measurement occasion than the trajectory of change that will be tested. Theories involving linear change need three time points, theories involving parabolic or U-shaped change need four time points, and so on. Additional time points are needed to test patterns that are expected to be autoregressive, periodic, or homeostatic—the very patterns evident (on a shorter time frame) in sleep and circadian rhythms. Finally, additional waves of data also increase statistical power and permit testing more complex time-dependent patterns. At a minimum, we recommend three time points of data for studies of change [60]. Alternatively, if feasibility prevents multiple time point measurements, three or more measures of each construct can be used to form cross-sectional composites that reduce measurement error.

### Timing matters: data must match the theory

Three additional elements are required for successful longitudinal change research designs [61, 77–79]. First, we must clarify the theoretical framework that underpins the research, and clearly link it to time. Next, the time course and extent of the data collection must match the theoretical framework being tested. Finally, the statistical models must be customized to appropriately test the theoretical framework by capitalizing on the repeated measures data collected [61, 80, 81].

#### What theoretical models of change are possible?

The first step in studying change is to identify a theory and link it to time [82]. It is possible to test multiple theories in a study, so long as the theory is identified in advance. This is because these theories—and their requirements for time—inform study design and measurement decisions, and these decisions require consideration before the launch of new data collection projects. These theories and measurement decisions also inform our ability to tease apart the potential hypothetical models of sleep and their sequelae as described above. Some theories may also be testable in existing archival or open science data sources, so long as the measurement frequency and timing match with the theories being tested. However, other theories and research questions might require de novo data collection in a new study.

Whether designing a new study or working with existing data, it becomes necessary to think differently about time. Depending on one’s design and/or research questions, time can be thought of as an outcome variable, as a predictor variable, or even as part of a dynamical system [80]. This alternative way of conceptualizing time contrasts with how many sleep researchers often regard time—as a design feature that is considered at the beginning of a study and then ignored or not analyzed. For example, many of us work to ensure that time is not a confounder in studies, either by controlling the measurement intervals (in experiments), or adjusting for (i.e. covarying out) time between the data collection intervals (in longitudinal studies). To study change, we need to embrace time as a valuable source of insight into the natural unfolding of events, causes, and directionality.

Some theories might suggest treating time as a predictor variable. For example, researchers interested in tying sleep to health will likely want to use statistical methods for analyzing change. These models use time as a predictor of some pattern in other variables, such as sleep and/or health. Some theories might propose one pattern of change (i.e. one trajectory) that fits all observations to varying degrees (i.e. with some amount of variability or heterogeneity), whereas other theories might posit that there are different patterns of change in different subgroups. With time as a consideration, we can describe the shape of this trajectory. For example, is the trajectory a straight line, a parabola (U-shaped), or some other function (like a staircase)? Next, once that trajectory is defined, we can determine whether there is variability or heterogeneity in those trajectories: the sample (overall) may be able to be defined using one trajectory, but at the individual level, certain people may not follow that exact trajectory. This introduces the possibility of evaluating predictors of the starting point (i.e. intercept, or level) and trend (i.e. slope, or rate of change) based on prior theory, empirical research, and clinical experience.

Other theories might suggest treating time as an outcome variable. For example, people interested in assessing transitions in and out of clinical status (e.g. developing a diagnosis, having clinically significant symptoms, achieving remission, or dying) will likely want to use statistical methods for event occurrence. These models predict the time some event occurs (i.e. the date or age at which someone “achieves” a status) from other time-invariant (stable) or time-varying (changing or modifiable) predictors in the study. Some theories might propose people “achieve” the status only once (i.e. they cannot move out of the status, as with death), whereas others might suggest people can move in and out of “having” the status (e.g. substance addiction); both of these types of models can be informative, so long as people can be precisely and reliably classified using meaningful units of time. Once the researcher decides how to treat status, they can then hypothesize about whether time-invariant and time-varying factors predict time-to-event.

A third group of models think of time as part of a dynamical system. These models recognize that there is some stability and/or homeostatic process inherent in the variables they have assessed across time. The goal of these models is to first identify and describe how these vectors of variables move throughout time and then use that information to infer whether patterns of change in one variable are temporally tied to patterns of change in a second variable. One extreme (and unlikely) example is when variables exhibit no change: in this illustrative example, all you would need to know to generate someone’s status in the future is their last status, because there is no movement in that variable across time. However, most variables exhibit some amount of carryover (stability) from one observation to the next, some amount of systematic (predictable) change, and some amount of measurement error. The researcher would therefore first model these dynamics (e.g. by considering lags or autoregressive covariance structures in the data) in at least two variables, and then afterwards would identify whether changes or perturbations in one variable precede the changes in another variable.

All things considered, a range of theoretical patterns of time-varying associations are possible, including designs beyond those described above. Time can be treated flexibly, with changes examined on the scale of minutes, to days, to measurement occasions, to years (and beyond). However, regardless of the theory used or the decision to treat time as a predictor or outcome (or both), scientists interested in studying change need to ensure there are more than enough appropriately spaced measurement occasions (time points), with reliable and meaningful assessment of time, to capture the theory they are interested in testing. Prior to launching any new data collection effort, we encourage considering the scaling of time, as well as the number of measurement occasions needed, and their timing and frequency [83, 84].

### Let’s get into the weeds: data analytic methods

#### Where does one start?

In considering methods of time-based data analyses, scientists can use methods that precisely test their hypotheses of interest. It is recommended to move away from the commonly used repeated measures analysis of variance (ANOVA) approach, as the time parameter in most repeated measures ANOVAs tests whether there is some difference somewhere in the outcome variable, but not whether the differences follow a particular pattern [77, 85]. This ambiguity is reflected in the omnibus nature of most time parameters in repeated measures ANOVAs with three or more time points. Because they have more than 1 *df*, they require multiple relatively lower-power post hoc follow-up tests to identify the time points of difference, from which time-relevant trends are often inferred. In addition to the power issues, these post hoc tests are also relatively imprecise tests of time, considering there is no single parameter that tells us whether the pattern of change is linear, quadratic, or some other pattern; instead, we just know there is a difference somewhere. Because a repeated measures ANOVA is unlikely to precisely test the focal hypotheses about time that likely generated a study, we encourage alternative approaches.

The good news is that several options for statistical designs exist that can be incorporated to more precisely study change. One particularly accessible approach that considers time as the predictor is the contrast analysis, which has analogs to the traditional *t*-test and repeated measures ANOVA [86]. Contrast analyses give scientists clear, precise information about the extent to which the observed pattern in the data matches the pattern implied by theory using easy-to-interpret effect sizes and significance tests. Additionally, competing theories can be simultaneously evaluated and compared against each other using these same significance tests and effect sizes [87]. Increased use of contrast analysis will allow sleep scientists to answer questions more precisely like “How good is this theory?” and “Which theory is better?” When considering change across time, repeated measures contrasts give focused, 1 *df* effect sizes and significance tests for questions like “How quickly do people improve?” and “Is improvement greater in group A relative to group B?” One bonus feature of contrast analyses is that they are simple enough to be calculated by hand using math adapted from *t*-tests and correlation coefficients. Easy-to-use scripts are also available for multiple statistical packages [86, 88].

#### What are more advanced methods to study change?

Other accessible models for questions about *pattern*, with time as the predictor variable, include hierarchical models. Fitting anything other than a flat line with zero slope and no heterogeneity (variance) means there is change. Once this change trajectory is fit, we can then incorporate time invariant (between subjects) and time varying (within subjects) predictors of this pattern. Common analytic approaches here include latent growth curve models, which can be estimated using multilevel modeling or structural equation modeling frameworks [89–92]. If different subgroups are expected to have different profiles of change, a growth mixture model or a latent profile analysis can be used [93–96]. These latter two approaches are similar to factor analysis; rather than identifying clusters of items in a factor with similar correlation structures (as in a factor analysis), these analyses identify clusters of people with similar change patterns.

Additional models for questions about shifts in and out of a particular status (e.g. having insomnia) include mixed models such as latent class transition analysis [97–100]. Latent class transition analyses are like the latent profile analysis described above, but they identify clusters of people with similar shifts in status across time. A second set of models that take a time-to-event approach, such as survival analysis [101, 102], may be more familiar to sleep scientists who study health data. These models typically estimate how much time elapses before some change in state or status, with time as the outcome variable. The name of the analysis implies prediction of death (i.e. “How long do people survive?” and “Is survival impacted by certain risk or protective factors?”). However, the approach is generalizable and useful for a number of time-varying outcomes, including age at diagnosis of a disease, completion of treatment (or drop-out), and even achievement of a particular score on a diagnostic or self-report inventory. Additionally, survival analytic methods can deal with events that occur along a non-parametric (i.e. not Gaussian normal or bell-curved) distribution, as well as events with censoring (e.g. when there is a lack of assessment or follow-up at the beginning or end of a study [103, 104]). For such analyses, a *life table* approach is a good place to start [105]. Once life table approaches are clear, other methods could be selected based on the features inherent in the data (e.g. censoring, the distribution of the events, and the nature of the predictors). Cox proportional hazards models are common in this area, but there are many alternatives, including the competing risks model [106–108], the accelerated failure time model [109–111], and others [105, 112–116]. Importantly, we suggest that sleep research should incorporate the study of change into these models. Rather than looking at a single time-invariant factor predicting the outcome (e.g. sleep at one time point predicting mortality risk), models should have repeated assessments of sleep and its sequelae (e.g. burden of chronic illness), in order to more clearly tease apart sleep’s relevance to public health.

A final option is to consider variables as part of vectors that may exhibit some stability (i.e. carry-over or inertia), some systematic change, and some error across time intervals. These analyses are sometimes labeled longitudinal panel models or cross-lagged models [66, 117–120], implying that the analyst is predicting some future status in one variable from that variable’s prior status (i.e. its own lag) and another variable’s prior status (i.e. the cross-lag). These types of models can answer questions like “Does yesterday’s sleep predict changes in today’s mood, accounting for stability in mood?” These models can be completed in several modeling frameworks, including structural equation modeling [121–123], multilevel modeling [124–126], autoregressive modeling [127, 128], and Granger causal modeling [129–133]. Several of these methods are regression-based, so many who are familiar with traditional ordinary least squares approaches may find these methods intuitive [134]. These methodologies are the most powerful with intensive, repeated measures data collection, but these methods are already commonly used in sleep science given that week-long sleep diaries and actigraphy are staples of our research designs [135, 136]. Sleep scientists may find many of these methods exciting because they can be adapted to formally test mediation, and they can be leveraged to answer questions at the between-person and within-person levels [107, 118, 126]. In other words, these can be used to study inter-individual change (i.e. typical “developmental” change across many people) and intra-individual change (i.e. person-specific change [65]). Thus, these methods can be used to help sleep scientists focus on which variables are best to intervene on for particular individuals and groups of people in clinical contexts.

### Spotlighting recent longitudinal sleep research studies

Excitingly, a growing body of research is investigating the evolving role of sleep across the lifespan. Here, we highlight a few key studies. In pediatric sleep research, substantial gaps remain in understanding the evolution of sleep patterns, behavior, and neurophysiology over time [137, 138]; however, ongoing efforts are addressing these gaps, as underscored in a recent review by Wang et al. [139]. In converging studies by McVeigh et al. [140] and Shimizu et al. [141], research on sleep problem trajectories during childhood reveals that individuals with persistent sleep problems experience poorer physical and mental health outcomes compared to those with consistently fewer sleep issues [140, 141]. Furthermore, early sleep not only influences a child’s mental health but also significantly affects maternal well-being. Gui et al. [142] recently published findings showing a bidirectional, longitudinal relationship between increased maternal depressive symptoms and persistent night wakings, observed from shortly after birth through 3 years of life. These studies help identify intervention targets that could ultimately foster better emotional, cognitive, and physical health development in both children and affected parents.

The adult sleep literature has also provided crucial insights into the profound relationship between sleep and health. Bowman et al. [143] leveraged the Study of Women’s Health Across the Nation (SWAN) (detailed in Table 2) to examine the long-term connection between depressive symptoms and sleep health outcomes in midlife women. In another study, Cavaillès et al. [145] investigated nocturnal sleep patterns over a span of 14 years, revealing that longer nocturnal sleep duration at multiple time points was associated with higher incidences of dementia. Furthermore, Tian et al. [146] analyzed 9-year sleep trajectories in older adults from the China Health and Retirement Longitudinal Study, revealing that individuals with stable, consistent nocturnal sleep patterns experienced healthier aging compared to those with shorter sleep patterns. Collectively, these long-term studies are crucial as they emphasize the impact of sleep on health outcomes, offering valuable insights into potential intervention opportunities and targets for optimizing health in older adulthood.

**Table 1:** Starter Steps Individual Scientists Can Take to Enhance Their Use of Longitudinal Methods for Studying Change

Number	Recommendation	Rationale
A priori research design decisions		
1	Include at least three measures of your construct of interest.	If your construct of interest is *time*, this will permit statistically separating measurement error from linear change. If your construct of interest is measured cross-sectionally, you can potentially form composites to minimize measurement error.
2	If time is the predictor, include at least one additional measurement beyond the pattern you are testing.	Additional waves of data permit more flexible modeling of time beyond a linear pattern. If *k* is the number of time points, there are *k* − 1 *df* available for testing models. Each time parameter requires additional *df* for testing (linear models require 1 *df*, quadratic models require 2 *df*, and so forth).
3	Consider the reliability and validity of measures.	Including measures with acceptable reliability and validity evidence will decrease measurement error and increase statistical power to detect change.
4	Plan to calculate several indices of time.	Measuring the day, month, and year of visit dates and birthdates will allow precise calculation of exact age and the spacing between measurement occasions.
5	Use an index of time that matches your theory.	Measuring time based on your theory is the only way to test that theory. For example, if you expect change with aging, your index of time should be chronological age.
6	Measure time precisely.	The model evaluates the effect of time based on the spacing across measurement occasions. Using precise measures of time (e.g. chronological age as an index of years and months since birth on the visit date, rather than a rounded year) reduces measurement error and simultaneously tells the model information about the spacing of assessments.
7	Make theory-informed decisions about the frequency and spacing of measurement occasions.	Measurement occasions need to occur when change is expected. Scientists should also consider the periodicity of their variables and ensure sufficient measurement occasions so patterns are not distorted (i.e. aliased).
Practical longitudinal research design considerations		
8	Measurement burst design.	Frequent measurements in a short period of time can provide additional data points to identify quick changes in shorter periods of time. For example, daily sleep diaries and cognitive tasks for 3 or more days in a row, three or more times in a year, can provide insight into changing patterns over time and reduce the burden on the participant.
9	Ecological momentary assessments (EMAs).	One way to increase the rate of measurement occasions within a shorter span of time can be EMAs (also known as event sampling). Data can be collected in multiple ways: (1) interval-contingent, i.e. specific predetermined points, such as morning and evening, or (2) signal-contingent, i.e. participants are randomly notified to respond to data questions at the time of notification, or (3) event-contingent, i.e. each time complete the data times participants complete data questions when a specific event occurs. For EMAs, multiple data collection points can occur within a day or across multiple days, and the timing between can differ across participants. Sampling at high frequencies can provide insight into fluctuating mood, thoughts, or sleep sequelae and the factors influencing their lability.
10	Collect at least three overlapping measures per domain.	In short, single day/night experimental designs, one can use multiple measures of a single domain (e.g. hippocampal-dependent memory). Using multiple measures can reduce the likelihood that a change score is an effect of error.
Post hoc research reporting decisions		
8	Report the reliability and validity of measures.	Reporting reliability and validity information will help clarify the extent of the confounding of change with measurement error.
9	Acknowledge confounds between age and cohort in your design.	Confounds can be addressed by recruiting additional cohorts into studies at later waves (i.e. using a cohort sequential design). When this is not feasible in cross-sectional studies and longitudinal studies—where age and cohort are confounded—acknowledgment of this limitation should be discussed.
10	Publicly archive your longitudinal data.	Archiving datasets with longitudinal methods will enhance our field’s ability to generate strong change-based conclusions about sleep and its sequelae. This archiving can be done in peer-reviewed journals or national repositories such as the National Sleep Research Resource (NSRR); datasets can be offered for use with or without authorship credit, depending on principal investigator (PI) preferences.
Enhance the ability of our field to conduct studies of change		
11	Leverage existing data sets with multiple assessments over time.	Large data sets provide the opportunity to investigate causal mechanisms for those unable to carry out longitudinal studies. Interested parties can often publish in these data sets, though each study has particular procedures for inquiring about data access and authorship. We encourage interested parties to contact PIs of the original study and invite them to be coauthors on projects which analyze their data in new and exciting ways.
12	Partner with organizations with electronic health records (EHRs) to obtain annual, standardized assessments in clinical populations.	Health systems are highly variable in what data are collected in a standard fashion. And unfortunately, sleep is rarely part of standardized question reporting. However, future agreements across major medical institutions could standardize the administration of sleep questionnaires in conjunction with other annually administered screeners, such as those for depression. This may foster the discovery of new relationships between sleep and health.
13	Consider the advantages of studies of change in sleep when peer-reviewing manuscript submissions and grants.	Encouraging authors to consider their ability to determine sleep’s role in consequential outcomes (whether spurious, causal, or due to reverse causation) will shift norms and incentivize longitudinal change-based analyses.

**Table 2:** Incentives the Field Should Leverage to Encourage Studies of Change

Create financial support for longitudinal change-based research		
1	Offer federal, foundation, and industry grants for longitudinal data archiving.	Most major funders now require public data archiving of research projects; additional grants or administrative supplements could be used for these purposes.
2	Increase the amount of federal, foundation, and industry grants dollars offered to studies which add additional time points.	Specific grants supporting longitudinal research can provide the fiduciary and infrastructure support required to undertake change-based research. The addition of multiple assessments increases the burden on research staff and participants alike; additional funds should be offered to incentivize these activities and offset their costs.
Create institutional and instructional support for longitudinal change-based research		
3	Create collaborative partnerships between researchers, medical providers, industry, and patients.	Clinical and industry partners may already be collecting longitudinal data in the form of EHR or wearables records. Creating infrastructure and financial incentives supporting these partnerships enhances our ability to make change-related conclusions in clinical populations and in private consumers.
4	Standardize graduate and post-graduate training in longitudinal research methods [144].	Currently, graduate methods training is piecemeal, and students may have limited opportunities based on what is available in their department or university. Familiarity with longitudinal data collection methods and statistical approaches should be a core competency in graduate training. Societies and funders can offer supplemental longitudinal methods training in the form of online webinars and summer schools for interested parties.
5	Incentivize additional training in longitudinal research methods and statistical approaches for faculty.	Current faculty may have limited training in longitudinal methods. Institutions, societies, and grant agencies can incentivize additional training by providing paid opportunities for summer school and administrative supplements for coursework and mentorship.
Reward longitudinal research		
6	Publish reports of newly available archival datasets. See below for publicly available datasets.^*^	Peer-reviewed journals can increase visibility and awareness of longitudinal change-based research by publishing standalone articles which detail publicly available datasets, the protocol(s) they come from, and how to access the data.
7	Create special issues dedicated to longitudinal research.	Journals can increase visibility and awareness of longitudinal change-based research by devoting special features to change-based methodology and findings.
8	Consider adopting minimum standards for reporting conclusions on aging and development.	Many studies confound measurement error, age, and cohort effects. Journals should encourage authors to report this as a limitation in discussion sections.
9	Count the collection and archiving of longitudinal datasets for grants, promotion, and tenure.	Similar to other research-based products and outputs, longitudinal change-based research efforts should be rewarded by counting them when PIs are applying for new opportunities.
^*^Publicly available large datasets (upon request)		
Websites with longitudinal publicly available longitudinal data sets: https://sleepresearchsociety.org/career-advancement/public-datasets/ https://sleepdata.org/ Pediatric sleep-large datasets Pediatric Adenotonsillectomy Trail of Snoring: This includes a large population of youth aged 3–12 years who are assessed at three visits, baseline, 6 and 12 months. This dataset includes a baseline polysomnography (PSG), and at all three visits, participants completed empirically validated questionnaires, neuropsychological tests, and biometrics were collected. Request for access: https://sleepdata.org/datasets/pats Rise & Shine: This dataset includes an initial, single prenatal visit, followed by five postnatal visits occurring between 1 and 24 months. Sleep patterns, actigraphy, questionnaires, social determinants of health, anthropometrics, gut microbiome were collected. Request for access: https://sleepdata.org/datasets/shine NCH Sleep DataBank: It includes a large set of pediatric patient sleep studies and longitudinal electronic medical record information. Almost 4000 pediatric patients are included, tracked for 2 years with electronic health record information. Request for access: https://physionet.org/content/nch-sleep/3.1.0/ Cleveland Family Study (CFS): It includes 361 families from diverse ethnic and racial backgrounds who are evaluated four times over 16 years. This data set includes in-home sleep studies to evaluate sleep apnea, metabolic, biometrics, and cardiovascular disease markers. Request for access: https://www.ncbi.nlm.nih.gov/projects/gap/cgi-bin/study.cgi?study_id=phs000284.v2.p1 Adult Wisconsin Sleep Cohort: Large, longitudinal data set evaluating adults every 4 years on sleep, anthropometrics, cardiovascular health, mental health, and mortality outcomes. Request for access: https://pophealth.wisc.edu/research/the-wisconsin-sleep-cohort/ All of Us Research Program: It is an NIH supported, diverse cohort of participants’ electronic medical record data, questionnaires, biospecimens, demographics, and social determinants of health. Data collection is ongoing with the goal of 1 million participants. Request for access: https://www.researchallofus.org/frequently-asked-questions/?gad_source=1&gclid=CjwKCAiAxKy5BhBbEiwAYiW---fvAQzru0gYhry_uAgjTiMrCrV4YXNDUCUP_-lLX-G48gMJxthcfBoCeAUQAvD_BwE Study of Women’s Health Across the Nation (SWAN): Large, longitudinal data collection in midlife women as they transition through menopause. Annual data are collected including anthropometrics, sleep, cardiovascular, cognitive, and psychosocial functioning. Data for baseline and 15 follow-up visits can be requested at: https://www.ncbi.nlm.nih.gov/projects/gap/cgi-bin/study.cgi?study_id=phs001470.v1.p1		

Looking to the future, digital health and wearable technology present exciting opportunities to track sleep-health associations across the lifespan [147]. These technologies have provided valuable insights into the long-term impacts of sleep on diverse populations. Recent highlights include tracking sleep before, during, and after pregnancy [148], examining the impact of sleep on college students’ mood during the COVID-19 pandemic [149], and exploring the interaction between sleep and chronic disease incidence through the All of Us Research Program [150]. A non-exhaustive list of publicly available datasets longitudinally tracking sleep and health is presented in Table 2.

### Zooming out: as a field, how can we set ourselves up for the study of change?

Using longitudinal designs is challenging, expensive, and requires a long time commitment from participants and research staff alike. These studies necessitate more personnel, lengthy participant engagement, and additional consideration of tasks and measures that will have similar reliability, validity, and measurement properties across repeated assessments, age groups, and cohorts. Practical constraints in feasibility, funding, and research infrastructures also reduce our opportunities to use longitudinal designs. Oftentimes, researchers must make the difficult decision as to whether to fund repeated measurements that include increased participant compensation, staff salaries, and material costs, or use that money to enhance the impact of shorter-term research, such as increased sample size, additional questionnaires, or increased recruitment efforts. Finally, competition for grants and academic job security encourages a publish or perish mentality, which can lead to “salami slicing” [151] and reduced public trust in our findings. Moreover, the incentive to run and publish the results of shorter cross-sectional studies can impede interest in pursuing longitudinal studies.

To support future change-focused research, we have identified several starting points and potential practical solutions (see Table 1) that sleep researchers can take to enhance their use of longitudinal methods for studying change.

Studying change is not just a job for sleep scientists: institutional support from funding agencies and academic institutions is necessary to shift norms and incentives in the field towards change-based research. The US National Institutes of Health (NIH) current mission statement is to “seek fundamental knowledge about the nature and behavior of living systems and the application of that knowledge to enhance health, lengthen life, and reduce illness and disability” [152]. This mission inherently underscores the need to assess change across the lifespan. Our societies and funding agencies must foster the space—and the financial resources—for scientists, allies, industry partners, and patients to engage in studies of change. Together, we can enhance our capacity to collect and analyze longitudinal data and amplify our ability to conclusively obtain an understanding of the role of sleep and its sequelae across the lifespan. See Table 2 for recommendations our field can take together.

## A Rejoinder: Change Matters. Let’s Go for It, Together

We, as sleep scientists, know the power of sleep. Together, we are working to unravel the complex relationships between sleep and health. We hope this article serves as a valuable resource for enhancing our research methods, improving the relevance of our findings for clinicians, educators, and policymakers, and translating those findings into practical applications in daily life. By integrating these elements into our work, we can better understand the critical role that sleep plays throughout the lifespan. With collaboration and the incorporation of even a few of these strategies, we can continue to deepen our understanding of sleep and its influence on health and development.

## Data Availability

No new data were generated or analyzed in support of this research. Note. Recommended references that are clear and serve as primers for these methods are indicated using the asterisks (***) below.
